# Gender Differences in Language of Standardized Letter of Evaluation Narratives in Osteopathic Emergency Medicine Residency Applicants

**DOI:** 10.7759/cureus.16622

**Published:** 2021-07-25

**Authors:** Justina Truong, Anthony Santarelli, Adam Dawson, John Ashurst

**Affiliations:** 1 Emergency Medicine, Kingman Regional Medical Center, Kingman, USA

**Keywords:** emergency medicine, assessment in health professions education, sloe, gender disparity, linguistic analysis

## Abstract

Background

The standardized letter of evaluation (SLOE) is used by emergency medicine (EM) faculty during the interview and match process. Data has shown that female allopathic applicants score higher in communal characteristics and have a greater number of ability words in the narrative portion of the SLOE as compared to their male counterparts.

Objective

To determine if there is a difference in the language used to describe male and female osteopathic applicants within the SLOE.

Methods

All applicants to a three-year EM residency within a single application cycle were eligible for inclusion. Exclusion criteria included allopathic applicants, applicants without a SLOE, or applicants with a SLOE only from the interviewing program. Data collected included applicant demographics and SLOE narratives. The previously validated Linguistic Inquiry and Word Count (LIWC; Pennebaker Conglomerates, Inc., Austin, TX) product was used to analyze word counts from the narrative portion of each SLOE. Descriptive statistics and t-tests for continuous data were used.

Results

Of the 577 applicants to the residency program, 318 met inclusion criteria and 33% were female. Females had a higher COMLEX-2 (590 vs 559; p=0.05) as compared to males but no difference was found for the remainder of the baseline demographics. No difference was found for the number of words in the narrative portion of the SLOE between males and females (males = 122 words; females = 127 words; p=0.53). Words within the social (p=0.006), achievement (p=0.007), and standout (p<0.001) categories were more frequent in osteopathic female applicants as compared to males. No statistical differences were detected for the other 13 categories analyzed.

Conclusion

In this sample of osteopathic applicants, little linguistic difference was noted for the narrative portion of the SLOE. SLOE authors did, however, use more social, achievement, and standout words to describe females as compared to male applicants.

## Introduction

The Council of Emergency Medicine Residency Directors (CORD) Standardized Letter of Evaluation (SLOE) has become the gold standard for letters of evaluation used by emergency medicine program directors during the application process [1]. First developed in 1995, the standardized letter of recommendation (SLOR) was developed as an evaluative tool to differentiate between a mass of applicants and assess clinical performance based upon direct and indirect observations in specific competencies that are important to the practice of emergency medicine [1-3]. In 2014 the name changed from SLOR to SLOE to better reflect that students were being evaluated and not always recommended for residency [1]. Recently, the SLOE has transitioned to an online format that includes both a summative and narrative evaluation [1].

A prior study from the narrative portion of letters of recommendation from those applying for chemistry and biochemistry positions has shown a difference in the words used to describe the different genders [4]. Similar to data seen in letters of recommendation, several key differences have been noted in the language used to describe female and male allopathic applicants to emergency medicine [5,6]. In these prior studies, words found within the categories of ability, social, and affiliation were more common in female applicants as compared to males [5,6]. However, no data is currently available for osteopathic applicants to emergency medicine because both of the prior linguistic studies excluded them from analysis [5,6]. The authors sought to determine if differences existed in the language used within the narrative portion of the SLOE for osteopathic applicants to emergency medicine residency. 

## Materials and methods

Setting

The emergency medicine residency at Kingman Regional Medical Center (KRMC) is a three-year program with 18 total residents who train in a rural community setting. Applications to the residency program are only accepted through the Electronic Residency Application Service (ERAS) and applicants must participate in the National Resident Matching Program (NRMP). Applications are accepted from all accredited medical schools. This study was approved by the institutional review board at KRMC. 

Study design

All applicants from the 2019-2020 application cycle to the KRMC emergency medicine residency were included in the sample. Exclusion criteria included applicants who held a terminal degree in allopathic medicine, applicants with an incomplete application at the time of download, applicants without a SLOE, or those applicants with only a SLOE from KRMC. Only the narrative portion of the SLOE was included in the final analysis. If applicants submitted more than one SLOE within the ERAS application, only narratives from the first rotation SLOE were considered. The narrative portion of the SLOE is limited to 350 words and asks the writer to address the following “(1) Areas that will require attention, (2) Any low rankings from the SLOE, and (3) Any relevant non-cognitive attributes such as leadership, compassion, positive attitude, professionalism, maturity, self-motivation, likelihood to go above and beyond, altruism, recognition of limits, conscientiousness, etc.” SLOE narratives were downloaded by authors and converted to Microsoft Word format for analysis. All stock sentences from the narrative portion of the SLOE about the rotation, institution, or how the grade was determined were removed prior to analysis. All data collection from the ERAS application was completed following submission of the institution's rank list to the NRMP. Data collected included applicant age at the time of application, gender, COMLEX 1 and 2 scores.

A linguistic approach similar to previous research using the Linguistic Inquiry and Word Count program (LIWC; Pennebaker Conglomerates, Inc. Austin, TX) was used to analyze the narrative portion of each SLOE [5,6]. LIWC is a validated text analysis program that compares words within a narrative to predefined word dictionaries with over 6,400 words [5,6]. Words are then broken down into 90 possible categories and the software reports the ratio of each word within a category to the total number of words with a given text file [5,6]. Based upon previous research, additional categories including grindstone traits, ability traits, standout adjectives, research terms, teaching terms, communal characteristics, and agentic characteristics were added to the predefined categories [4-6]. 

Statistical analysis

As previous work on the topic has illustrated that predefined LIWC categories appear more commonly among female allopathic students, a one-tailed apriori power analysis was completed to determine the number of subjects needed to detect a difference between unitary LIWC categories [5,6]. As previous differences were detected from sample sizes of 200 to 300 applicants, a large effect size of 0.3 was estimated [5,6]. With an estimated allocation ratio of males to females of 1.5, an estimated 80% power would be needed to detect gender differences on single LIWC categories with a total sample of 302. All a priori analysis was conducted using the G*Power software (University of Dusseldorf, Germany) and all post-hoc analysis was conducted using IBM SPSS statistics version 27 (IBM Corp., Armonk NY). 

Differences among the characteristics of male and female applicants were assessed using descriptive statistics. The Mann-Whitney U test was used to assess significance among the ages and COMLEX scores. Differences between the narrative portion of LIWC categories among male and female applicants SLOEs were assessed using the Wilcoxon 2-sample t-test. 

## Results

There was a total of 577 applications to the KRMC emergency medicine residency during the 2019-2020 application. Of the 318 applicants from osteopathic programs, 67% (213/318) were male and 33% (105/318) were female. Male and female applicants were of the same age (p=0.15) and scored similar values on the COMLEX-1 (p=0.67). However, female applicants outperformed male applicants on the COMLEX-2 (p=0.05) (Table 1). 

**Table 1:** Baseline demographics of osteopathic applicants Data were presented as median and interquartile ranges

Applicant Information				
Variable	Total (n = 318)	Male (n = 213)	Female (n = 105)	p-value
Age	29.0 (28 - 31)	29.0 (28 - 31)	29.0 (27 - 31)	0.15
COMLEX-1	533 (488 - 588.8)	534 (488 - 589)	528 (488 - 586)	0.67
COMLEX-2	562 (510.5 - 614.5)	559 (508 - 611)	590 (512 - 632)	0.05

Analysis of the narrative portion of SLOE’s with LIWC revealed a similar evaluation median word length for both genders (males = 122 words; females = 127 words; p=0.53) and each sentence had a median length of 15.2 words per sentence, which did not vary by gender of the applicant (males = 15.3 words; females = 14.7 words; p=0.53). Following analysis within the LIWC dictionaries, the frequency of words referencing the categories of social (p=0.006), achievement (p=0.007), and standout (p<0.001) all varied by gender with females receiving a higher frequency of descriptors than males in each category (Table 2). There was no gender-based difference among the remaining 13 categories evaluated.

**Table 2:** Selected LIWC (Linguistic Inquiry and Word Count) output variables for osteopathic emergency medicine applicants Data were reported as median and median and interquartile ranges (IQR).

Variable	Total N=318 (IQR)	Male n=213 (IQR)	Female n=105 (IQR)	p-value
Word count	122 (85 - 165.5)	122 (84 - 161)	127 (87 - 177)	0.53
Words per sentence	15.2 (12.7 - 17.8)	15.3 (12.7 - 17.9)	14.7 (12.7 - 17.7)	0.53
Affect	7.1 (5.7 - 9)	7.1 (5.6 - 8.8)	7.3 (5.9 - 9.3)	0.25
Positive	6.4 (5 - 8.3)	6.3 (4.8 - 8.3)	6.8 (5.4 - 8.4)	0.30
Negative	0 (0 - 1.0)	0 (0 - 0.9)	0.4 (0 - 1)	0.30
Social	12.2 (10.3 - 14.3)	12.0 (9.9 - 13.9)	13.0 (10.4 - 15.2)	0.006
Cognitive process	8.6 (6.7 - 10.9)	8.6 (6.6 - 11.4)	8.8 (6.8 - 10.6)	0.83
Affiliation	2.6 (1.4 - 3.9)	2.4 (1.3 - 3.9)	3.1 (1.5 - 4.0)	0.16
Achievement	4.6 (3.4 - 6.4)	4.6 (3.2 - 6)	5.2 (3.8 - 7)	0.007
Power	3.7 (2.6 - 5)	3.7 (2.4 - 5)	3.8 (2.7 - 5.2)	0.49
Reward	2.7 (1.6 - 3.7)	2.7 (1.5 - 4)	2.7 (1.7 - 3.5)	0.96
Risk	0 (0 - 0.5)	0 (0 - 0.5)	0 (0 - 0.4)	0.66
Standout	0 (0 - 1.2)	0 (0 - 1.1)	0.8 (0 - 1.5)	0.000
Ability	0.7 (0 - 1.3)	0.7 (0 - 1.4)	0.7 (0 - 1.3)	0.74
Grindstone	1.4 (0.7 - 2.3)	1.4 (0.7 - 2.3)	1.5 (0.6 - 2.3)	0.97
Teaching	1.5 (0.9 - 2.3)	1.5 (0.9 - 2.3)	1.5 (0.9 - 2.4)	0.98
Research	0 (0 - 0.3)	0 (0 - 0.5)	0 (0.0-0.0)	0.52
Communal	0 (0.0-0.0)	0 (0.0-0.0)	0 (0.0-0.0)	0.63

## Discussion

Previously, a difference in the words used to describe male and female allopathic applicants to emergency medicine was noted in the narrative portion of the SLOE where females had a higher frequency of words in the social, affiliation, and ability categories [5,6]. When the two previous studies were compared, both found a statistically significant difference in the number of ability words used to describe female allopathic applicants as compared to their male counterparts [5,6]. Although these findings were not replicated by the current study, the authors were able to replicate the finding that those words used in the social category among female osteopathic applicants were more likely than their male counterparts. This data coincides with other studies showing that women applying for residency positions are more likely to be described by non-ability attributes [7-9].

This study, however, contradicts previous research and shows that SLOE writers for osteopathic females use more standout and achievement words in the narrative portion of the SLOE as compared to males. The two previous studies directly examining this topic in allopathic applicants found no difference in these categories [5,6]. This could be related to the female applicants in the study scoring higher on the COMLEX-2 as compared to their male counterparts. Previous data has also shown that allopathic female applicants had better composite scores, comparative rank scores, and rank list position scores as compared to their male counterparts [10]. Although not directly studied, these results may coincide with better quantitative scores achieved by osteopathic female applicants on the SLOE as compared to males and cause SLOE authors to use more standout and achievement words in the narrative portion. Further studies will be needed to corroborate this theory and the current findings.

Despite these small word differences in the standout, achievement, and social category, the remainder of the 13 categories assessed by the linguistic analysis showed no statistical differences. This data coincides with the previous research on the topic and shows that SLOE writers as a whole describe male and female osteopathic applicants using similar language [5,6]. This could be in part due to restrictions on the number of words used in each SLOE and the instructions for authors of the SLOE. Further research should focus on the correlation of the narrative portion of each SLOE and how it relates to an applicant's clinical years once matched into residency. Research should also focus on determining if any differences exist in the language used in the SLOE based upon the writer's gender. 

Limitations

This was an analysis of a single program’s applicants during one application cycle and only osteopathic applicants were included in the analysis. Examining all applicants from this cycle may have altered the results. Much like previous research on the topic, pre-defined dictionaries were also used to analyze the narrative portion of each SLOE. Although these have been used previously in other research on this topic, the dictionaries may not include all words that are important for each category. When very few words were used within a single category, a statistical difference could be noted between the two genders. It is unclear, however, if this statistical difference correlates to the clinical acumen of the candidate. 

## Conclusions

During a single application cycle at a community emergency medicine residency, linguistic analysis noted differences in the language used to describe female and male osteopathic applicants in the categories of social, achievement, and standout words. However, no difference was noted between the genders in the remainder of the categories evaluated in the narrative portion of the SLOE. 
